# Gendering Islamophobia, racism and White supremacy: Gendered violence against those who look Muslim

**DOI:** 10.1177/2043820616655018

**Published:** 2016-07-22

**Authors:** Peter Hopkins

**Affiliations:** Newcastle University, UK

**Keywords:** anti-Muslim, hate crime, Islamophobia, masculinity, racism, violence

## Abstract

Defendants in both racist and religiously motivated hate crimes in the United Kingdom are usually White men, with these incidents tending to take place in public spaces, especially those close to religious and community buildings. Focusing on the experiences of Muslim men and women in the United Kingdom – including others who *look* Muslim – I explore common forms of religiously and racially motivated violence and argue that these constitute gendered violence. Policies targeted at key groups who are victims and perpetrators of religiously and racially motivated violence ignore the issue of violence and fail to acknowledge its gendered nature.

In this piece, I argue that common forms of religiously and racially motivated violence in the United Kingdom constitute gendered violence. These forms of gendered violence are especially targeted at Muslims; the perpetrators are overwhelmingly White men and the victims are women but also men who are perceived to follow the Islamic faith. I draw attention to the intersection of gendered violence with other axes of difference including race, masculinity and place to explore the gendering of Islamophobia and racism. In developing strategies to tackle these issues, it is crucial to recognize the ways in which gendered violence is shaped by gendered identities and stereotypes. Here, I focus upon the experiences of Muslim men and women in the United Kingdom, including those who may be mistaken for being Muslim as a result of the complex ways in which their style of dress, skin colour or other phenotypical features – mostly associated with processes of embodiment (Longhurst and Johnston, 2014) – results in them being associated with the Islamic faith and targets of gendered Islamophobic abuse and physical assault. Blackwood et al. (2013: 1097) note in their research with Muslims that ‘all those who talked about airport [security] encounters were explicit that the basis of their treatment was that they were Muslims and that anyone who was Muslim (or fitted a Muslim stereotype) was open to similar treatment’ (Blackwood et al., 2013: 1097). Here, the everyday gendered, embodied and emotional encounters across racialized and religious differences connect with broader geopolitical debates and speak to advancing feminist geopolitics, and in particular, ‘an intimate critical geopolitics of the everyday’ (Cowen and Story, 2013: 353).

These observations emerge from a context in the United Kingdom where there has been an increasing presence of right-wing groups that have undoubtedly been bolstered by ongoing experiences of austerity. In early 2009, emerging from the English football supporters masculine scene, the English Defence League’s (EDL) ‘vocal opposition to what it calls “militant Islam” appears to have garnered considerable support from marginalized and disadvantaged white working class communities’ (Treadmill and Garland, 2011: 621). The EDL – and its sister, the Scottish Defence League – regularly hold street protests in cities across the United Kingdom. In addition to such groups, there also exist smaller, sometimes shorter term, localized right-wing groups. Recent reports about Islamophobia and anti-Muslim hate crime in the United Kingdom point to the ways in which the actions of those connected with such right-wing groups – as well as those who engage in acts of religious and racial hatred without necessarily having a group affiliation – result in persistently unjust, regularly violent and relentlessly unpleasant verbal, physical and emotional attacks against Muslims and those who are perceived to be Muslim. These anti-Muslim attacks range from murder, serious assaults and arson through to spitting, threatening and abusive words or behaviour (Copsey et al., 2013; Lambert and Githens-Mazer, 2010). One of the many problematic aspects of the existence of such right-wing groups is that they tend to be dominated by White men who enact specific practices associated with masculinity and White supremacy, with victims being women and men who are perceived to follow the Islamic faith. I therefore recast this as a form of gendered violence that is energized by ongoing debates about race and ethnicity, Islamophobia, multiculturalism, citizenship and belonging. This is not only racist violence as it is inflected with gender and bolstered by sexism, patriarchy and White supremacy (Hopkins et al., 2015).

A recent report about *Anti-Muslim Hate Crime and the Far Right* (Copsey et al., 2013) uses figures from the Crown Prosecution Service in the United Kingdom in 2011–2012 to show that the majority of defendants in both racist and religiously motivated hate crimes were men (83%), with most being White British (73.6%) and aged 25–59 (54.2%). Just under 50% involved offences against the person and 32.5% were public order offences. The primary sites of these reported hate crimes tended to be in public spaces (such as parks, streets, footpaths) although the report comments on the likelihood that these are occurring in the vicinity of religious and community buildings. Clearly, such statistics need to be approached with caution; as a result of under-reporting, hate crime is not well understood by the public, and there are indications that over 50% of hate crime incidents go unreported so many will suffer in silence (Copsey et al., 2013). Incidents may be unreported due to lack of confidence in the justice system, assumptions that the police would not be interested, or the frequency of incidents resulting in non- or under-reporting.

A small proportion of the perpetrators of hate crime in Britain belong to far right groups, though the majority are ‘characterised by their very ordinariness’ (Copsey et al., 2013: 11). However, those committed by far right extremists are likely to be more extreme and premeditated than those committed by ‘ordinary people’. With regard to cases of religious hate crime and in situations where the religion of the victim was known, 52% (or 632) of incidents were against Muslims (26% were against Jews and 14% against Christians). As this report makes clear, ‘when it comes to hate crime, most victims are Muslim’ (Copsey et al., 2013: 7). Of the 150 incidents reported through the Tell Measuring Anti-Muslim Attacks project between 1 April 2012 and 30 April 2013, 12% involved assault, 12% involved property damage, 11% involved the distribution of anti-Muslim literature and 8% were noted as involving ‘extreme violence’ (Copsey et al., 2013: 15). The gender breakdown shows, again, that just under 80% of the perpetrators of offline incidents were men and most were under the age of 40, with men being responsible for 82% of online incidents. In the case of offline offences, the majority of the victims were women, particularly those who were identifiable as Muslim. The majority of online incidents involved the threat of participating in some form of offline action. Of the online incidents, 70% report a link to the far right (Copsey et al., 2013). The online presence of racist groups continues to cause concern; the Internet acts as an important communication tool for White supremacist, sexist and Islamophobic groups and individuals. These all point to the diversity of sites at which such racist and religious hatred can be promoted, and the worrying tendency for this to be shaped by sexist, patriarchal and chauvinistic values and behaviours. Furthermore, we see here the gendering of both the perpetrators and the victims of racially and religiously motivated violence as the first is motivated by racism that is partly shaped by patriarchy and sexism with the victims being targeted as a result of these racist interpretations of the intersections between gender, ethnicity and religion.

Lambert and Githens-Mazer (2010) produced a substantial report about Islamophobia and anti-Muslim Hate Crime: UK Case Studies 2010, noting that a key motivation behind the increasing numbers of anti-Muslim attacks is the politics of groups such as the British National Party (BNP) and the EDL. They refer to a ‘violent extremist nationalist milieu’ (Lambert and Githens-Mazer, 2010: 33) that is exacerbated by the negative influence of mainstream politicians (the hard line approach to immigration by many of the main political parties being one such example). Furthermore, although most of those who commit anti-Muslim hate crimes are not members of the BNP or EDL, these people have become increasingly angered and convinced by the portrayal of Muslims in the media as extremists and a threat to national security. Some key points discussed by Lambert and Githens-Mazer (2010) include the increasing number of EDL events that provoke fear and intimidation within Muslim communities and that many racist groups have refocused their aims to specifically target Muslims (some gangs, for example, previously not involved in racist activities, are now targeting Muslims). Victims, they observe, are of a ‘discernable and distinctive Muslim appearance’ (Lambert and Githens-Mazer, 2010: 48), and that while victims of racist attacks were invariably men, victims of anti-Muslim attacks are often women. The report found that a disturbing number of Muslim women who wear a niqab, hijab or burka were the victims of anti-Muslim hate crimes. These tend to happen in public spaces, on trains, buses, shopping centres and so on – often when other people were there yet did not intervene. Attacks against ‘identifiable Muslims’ (Lambert and Githens-Mazer, 2010: 35) tended to take place near mosques or ‘against Muslims wearing Islamic clothes and, in the case of men, Islamic beards or, in the case of women, hijabs, niqabs or burkas’.

The dominant polices that are targeted at both Muslims and young White men erase the issues explored above (including the gendering of Islamophobia and racism) and instead, exacerbate already existing gendered, racial and religious inequalities and injustices. On the one hand, recent policies targeted at the Muslim community in the United Kingdom have focused on counterterrorism measures and the prevention of violent extremism. The impact of such policies has been well documented in the literature, with concerns expressed about how they construct Muslims as potentially threatening people who have the capacity to engage in extremist behaviours in different places (e.g. Hopkins, 2011, Spalek and McDonald, 2009). Choudhury and Fenwick (2011) note that stop and search under section 44 of the Terrorism Act became the main mechanism through which young Muslim men engaged with the police, observing that since 2001, over half a million stop and searches had taken place but none had led to any convictions in relation to terrorism. Such procedures securitize, stigmatize and marginalize those of Muslim appearance (Millings, 2013), especially Muslim men but also Muslim women, and give additional justification to those with White supremacist and patriarchal views.

On the other hand, from 1997 a key policy targeted particularly (but not only) at White working-class youth in the United Kingdom was about the label of ‘young people not in education employment or training (i.e. NEET)’; this ‘became the key youth policy of the New Labour government’ (MacDonald, 2011: 430) and arguably continued to be an important focus of the Conservative and Liberal Democrat coalition government too. This policy sees the problem with White working-class youth as being about education and employment only and fails to engage with the ways in which some White working-class young men engage in sexist, racist, White supremacist actions and behaviours (without necessarily being aware of the illegal nature of many of these) and therefore neglects to offer strategies for the prevention of sexism, racism, religious intolerance and White supremacy among young people.

Both of these examples of policies targeted at the key groups who are victims and perpetrators of religiously and racially motivated violence ignore the issue of violence and fail to acknowledge its gendered nature. Meanwhile, mainstream debates about gendered violence almost exclusively cast it as a problem existing within ethnic or religious groups, rather than across them, therefore overlooking the relational nature of this phenomenon. Recognizing that gendered violence – a growing and horrific phenomenon in the United Kingdom – is profoundly shaped by gendered identities and stereotypes, is crucial to developing strategies to help tackle it.
